# Effects of food and ethnicity on the pharmacokinetics of venadaparib, a next-generation PARP inhibitor, in healthy Korean, Caucasian, and Chinese male subjects

**DOI:** 10.1007/s10637-023-01405-z

**Published:** 2023-12-15

**Authors:** Hyun Chul Kim, Eunsol Yang, Soyoung Lee, Jaeseong Oh, Myongjae Lee, ChaeEun Lee, Kyoung Soo Ha, Won Sik Lee, In-Jin Jang, Kyung-Sang Yu

**Affiliations:** 1 Department of Clinical Pharmacology and Therapeutics, Seoul National University College of Medicine and Hospital, 101, Daehak-ro, Jongno-gu, Seoul, 03080 Republic of Korea; 2 Integrated Major in Innovative Medical Science, Seoul National University Graduate School, Seoul, Republic of Korea; 3 Present Address: Department of Bioengineering and Therapeutic Sciences, University of California, San Francisco, San Francisco, CA United States of America; 4 Kidney Research Institute, Seoul National University Medical Research Center, Seoul, Republic of Korea; 5 Present Address: Department of Pharmacology, Jeju National University College of Medicine, Jeju, Republic of Korea; 6 Idience Co., Ltd., Seoul, Republic of Korea; 7 Idience Inc., Irvine, CA 92618 USA

**Keywords:** Food effect, Ethnic effect, Venadaparib, PARP inhibitor, Pharmacokinetics

## Abstract

**Aim:**

Venadaparib is a next-generation poly(ADP-ribose) polymerase inhibitor under development for treating gastric cancer. This study aimed to evaluate the effects of food and ethnicity on the pharmacokinetics (PKs) and safety of venadaparib after a single oral administration in healthy Korean, Caucasian, and Chinese male subjects.

**Methods:**

In this randomized, open-label, single-dose, two-sequence, two-period, and crossover study, Korean and Caucasian subjects received venadaparib 80 mg in each period (fasted or fed state) with a seven-day washout. In an open-label, single-dose study, Chinese subjects received venadaparib 80 mg only in the fasted state. Serial blood samples were collected up to 72 h post-dosing.

**Results:**

Twelve subjects from each ethnic group completed the study. The geometric mean ratios (90% confidence intervals) of the maximum plasma concentration (C_max_) and area under the plasma concentration-time curve from time zero to the last measurable time point (AUC_last_) of venadaparib for the fed to fasted state were 0.82 (0.7457–0.9094) and 1.02 (0.9088–1.1339) in Koreans, and 0.77 (0.6871–0.8609) and 0.96 (0.9017–1.0186) in Caucasians, respectively. No statistically significant differences were observed in C_max_ (*P*-value = 0.45) or AUC_last_ (*P*-value = 0.30) among the three ethnic groups. A single venadaparib dose was well-tolerated.

**Conclusion:**

The overall systemic exposure of venadaparib was not affected by the high-fat meal, despite delayed absorption with a decreased C_max_ in the fed state. The PK profiles were comparable among the Korean, Caucasian, and Chinese subjects. A single venadaparib 80 mg dose was safe and well-tolerated in both fasted and fed states.

**Supplementary Information:**

The online version contains supplementary material available at 10.1007/s10637-023-01405-z.

## Introduction

DNA has a relatively stable structure; however, it is constantly damaged by endogenous and exogenous agents [1, 2]. Poly(ADP-ribose) polymerases (PARPs), specifically PARP1 and PARP2, are the main enzymes for repairing single-strand breaks in DNA through the base-excision repair pathway [1, 3, 4]. Under PARP inhibition, single-strand breaks in DNA are further damaged to double-strand breaks, which can be repaired by homologous recombination mechanisms in normal cells, whereas the double-strand breaks cannot be properly repaired in cancer cells with mutations in breast cancer susceptibility genes (*BRCA1* and *BRCA2*), leading to cell death [3, 5]. Therefore, PARP1 and PARP2 inhibition provides cancer-targeted potency by disrupting the DNA repair processes in cancer cells [6].

To date, four PARP inhibitors (olaparib, rucaparib, niraparib, and talazoparib) have been approved by the United States Food and Drug Administration (FDA). Each PARP inhibitor treats different cancers according to its indication but targets cancer cells with homologous recombination deficiency or deleterious *BRCA* mutations. In addition to the approved indications, ongoing clinical and non-clinical studies have shown that PARP inhibitors can be used as monotherapies or in combination with other therapies in several cancers or non-oncological areas [7–9].

Venadaparib, known as IDX-1197 or NOV140101, is a next-generation PARP inhibitor under development to treat various solid cancers by Idience Co., Ltd., Seoul, Republic of Korea. In terms of PARP enzyme selectivity, compared with those of olaparib, the in-vitro inhibitory concentration at 50% of the total effect (IC_50_) values of venadaparib were similar for PARP1 and PARP2; however, higher for other PARPs, especially PARP3 and PARP5 [10]. Additionally, the activity of inhibiting poly(ADP-ribose) formation or trapping PARP was comparable with that of talazoparib [10–12]. The antitumor potency of venadaparib was 40–440-fold higher than that of olaparib in cancer cell lines with *BRCA* mutations, and the tumor growth inhibition rate was higher than that of olaparib in a patient-derived xenograft mouse model (131.0–135.2% vs. 118.2%), with a greater safety margin [10].

To date, a phase 1 study (NCT03317743) evaluating the safety, tolerability, pharmacokinetics (PKs), pharmacodynamics (PDs), and anticancer efficacy of venadaparib has been completed in Korean patients with advanced solid tumors. In this study, multiple three-week venadaparib administrations were safe and well-tolerated in the dose range of 2–240 mg/day. Systemic exposure to venadaparib showed a dose-proportional relationship within this dose range. The maximum plasma concentration (C_max_) of venadaparib was reached after approximately 2 h, and the mean elimination half-life (t_1/2_) ranged from 7 to 13 h (data on file). Additional clinical trials of venadaparib are ongoing in patients with homologous recombination repair-mutated solid tumors (NCT04174716) and advanced gastric cancer (NCT04725994).

During the early phase of drug development, assessing food-drug interactions and ethnic differences in drug exposure and response is crucial, and the results will help in conducting further patient studies and establishing drug labeling [13, 14]. Therefore, we aimed to evaluate the effect of food on the PKs, safety, and tolerability of venadaparib after a single oral dose in healthy Korean and Caucasian male subjects. We also explored the effect of ethnicity on venadaparib among Korean, Caucasian, and Chinese subjects.

## Methods

The Korean Ministry of Food and Drug Safety and the Institutional Review Board of the Seoul National University Hospital reviewed and approved this study. The study was registered in the open registry of ClinicalTrials.gov (NCT05202912) and conducted following the Declaration of Helsinki and Korean Good Clinical Practice guidelines [15, 16]. Written informed consent was obtained from all subjects prior to any procedures.

### Subjects

Healthy Korean, Caucasian, and Chinese male subjects aged 19–50 years with a body weight of ≥ 55.0 kg and a body mass index of 18–30 kg/m^2^ were eligible for this study. The Korean subjects had to be born in Korea, have biological parents and grandparents of Korean origin, and had lived outside Korea for less than 10 years. The Caucasian subjects had to be born in Europe, have biological parents and grandparents of European origin, and had lived outside Europe for less than 10 years. The Chinese subjects had to be born in China, have biological parents and grandparents of Chinese origin, and had lived outside China for less than 10 years. The major exclusion criteria were as follows [17]: any history of clinically significant disorders; hemoglobin level of < 12.0 g/dL; estimated glomerular filtration rate based on the modification of the diet in the renal disease formula of < 60 mL/minute/1.73 m^2^; Bazett’s corrected QT interval (QTcB) of > 450 milliseconds; subjects who had used drugs that induce or inhibit drug-metabolizing enzymes within 1 month prior to the first dose of study drug; and subjects who had consumed foods containing grapefruit within 24 h of each admission. Moreover, subjects or their partners should have used medically acceptable contraception for at least three months after the last venadaparib dose [18, 19].

### Study design

**Fig. 1:**
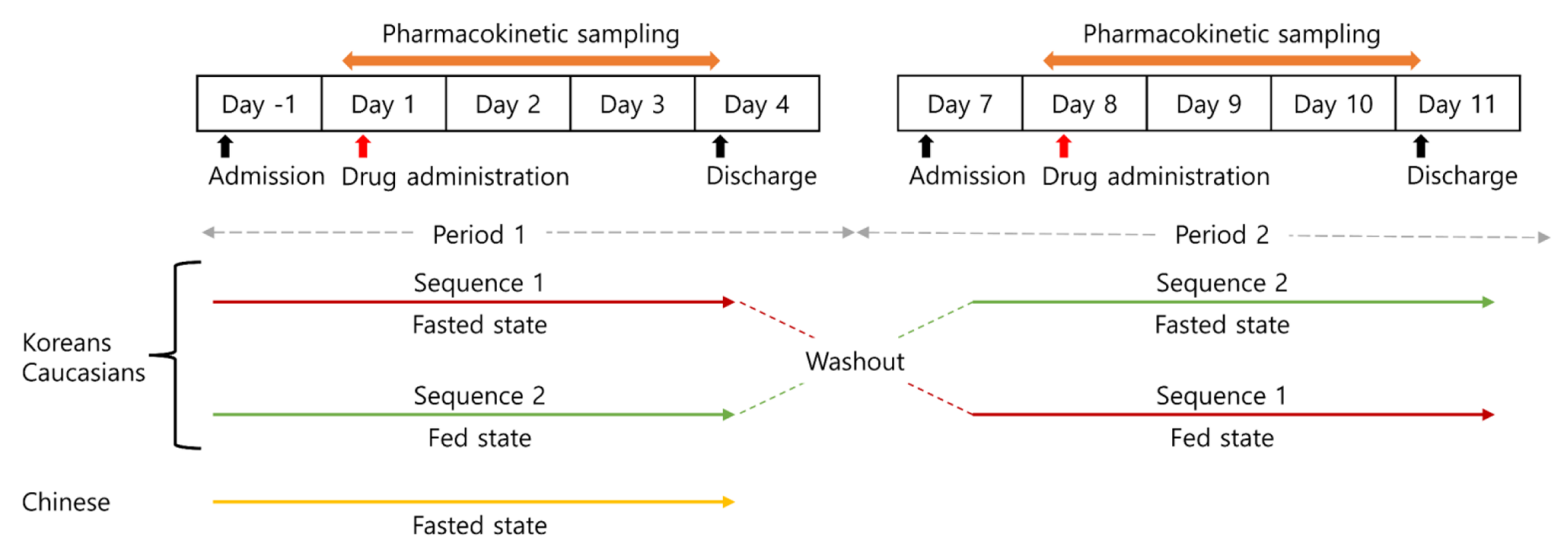
Study design

A randomized, open-label, single-dose, two-sequence, and two-period crossover study was conducted with 12 Korean and 12 Caucasian subjects. Subjects in each ethnic group were randomly assigned to one of the two sequences at a ratio of 1:1 (Fig. 1). The subjects were orally administered 80 mg of venadaparib in each treatment period (fasted or fed state), following an overnight fast of at least 10 h. There was a seven-day washout between the two treatment periods. In the fed state, the subjects consumed an entire high-fat meal (total of 800–1000 kcal, fat of 500–600 kcal) within 20 min and were subsequently administered the study drug with 240 mL of water 30 min after the start of the meal [20]. An open-label, single-dose study was conducted on 12 Chinese subjects who received 80 mg of venadaparib only in the fasted state (Fig. 1).

Based on the PK results of venadaparib in a previous study (NCT03317743), serial blood samples for the PK analysis of venadaparib were collected at 0 (pre-dose), 0.25, 0.5, 1, 2, 3, 4, 6, 8, 10, 12, 24, 36, 48, and 72 h post-dose for all treatment and ethnic groups. The blood samples were collected in a K_2_-ethylenediaminetetraacetic acid (EDTA) vacutainer, centrifuged at 4 ℃ and 3000 rpm for 10 min, and stored at − 70 ℃ until analysis.

### Determination of plasma venadaparib concentration

Plasma concentrations of venadaparib were determined using validated high-performance liquid chromatography (Exion LC, AB Sciex) coupled with mass spectrometry (Triple Quad 5500+, AB Sciex). Venadaparib as an analyte and olaparib as the internal standard were separated using a Gemini 3 μm NX-C18 110 Å (100 × 3 mm) column (Phenomenex, CA, USA) at 40 ℃. The mobile phase comprised 0.1% formic acid in distilled water and 100% acetonitrile at a flow rate of 0.3 mL/minute. Venadaparib and olaparib were detected with a turbo-ion spray in positive ionization mode at an m/z transition of 407.2 → 70.0 and 435.2 → 281.1, respectively [21].

The calibration curve for venadaparib ranged from 5 to 5000 μg/L, with precision and accuracy ranging from 1.89 to 4.00% and 92.98 to 102.80%, respectively. For the quality control sample data of venadaparib, the precision and accuracy ranged from 4.38 to 5.18% and 96.75 to 102.59%, respectively.

### Pharmacokinetic and statistical analyses

The PK parameters of venadaparib were calculated by a non-compartmental method using Phoenix WinNonlin software version 8.3.4 (Certara, NJ, USA). The area under the plasma concentration-time curve (AUC) was determined using the linear-up log-down trapezoidal method. The primary PK parameters were C_max_ and AUC from time zero to the last measurable time point (AUC_last_) for comparison between the exposures to the effects of food and ethnicity. The following secondary PK parameters were calculated: AUC from time zero to infinity (AUC_inf_), time to reach C_max_ (T_max_), t_1/2_, apparent total clearance (CL/F), and apparent volume of distribution (V_d_/F).

Statistical analyses were conducted using SAS software version 9.4 (SAS Institute Inc., NC, USA). The primary PK parameters in the fed and fasted states were compared by estimating the geometric mean ratios and 90% confidence intervals using a linear mixed-effects model. The model included the sequence, period, and treatment as fixed effects, and the subject nested within the sequence as a random effect. Analysis of variance (ANOVA) tests were conducted to assess possible differences in the values of the primary PK parameters among the ethnic groups in the fasted state.

### Safety assessment

Safety was assessed in all subjects who received the study drug at least once throughout the study based on adverse event (AE) monitoring, physical examinations, vital signs, 12-lead electrocardiograms (ECGs), and clinical laboratory tests.

To support the evaluation of the relationship between concentration and QT/corrected QT (QTc) interval effects in the early stage of the clinical development of venadaparib, triplicate ECG tests were conducted in the middle of the study [22]. A triplicate ECG test was conducted in three Caucasian and five Chinese subjects after the protocol amendment. The 12-lead ECG schedules at 1, 2, 3, 4, 6, 8, 10, 12, 24, and 48 h post-dosing were added to the existing time points at 0 h (pre-dosing) and 72 h post-dosing. Fridericia’s corrected QT interval (QTcF) was collected, along with the existing ventricular rate, PR interval, QRS duration, QT interval, and QTcB. Triplicate ECGs were measured three times at intervals of 30 s to 2 min at each time point after at least 5 min of resting, and the median value of the three measurements was considered a representative value. QTcF and QTcB were calculated using the following population-derived correction equations: QTcF = QT/RR^0.33^ and QTcB = QT/RR^0.5^ [22].

## Results

### Study population

Thirty-seven subjects were enrolled, and twelve Korean, twelve Caucasian, and twelve Chinese participants completed the study. One Korean subject withdrew consent for personal reasons before completing the first fed period.

The mean ± standard deviation values of demographic characteristics of the enrolled subjects were as follows: age of 31.11 ± 7.20 years, height of 174.05 ± 5.85 cm, weight of 75.94 ± 11.54 kg, and body mass index of 25.00 ± 3.10 kg/m^2^. The three ethnic groups showed statistically significant differences in age (*P*-value = 0.03) and height (*P*-value < 0.01) (Table 1).

**Table 1:** Comparison of demographic characteristics between Korean, Caucasian, and Chinese subjects

Characteristics	Korean (N = 13)	Caucasian (N = 12)	Chinese (N = 12)	*P-*value*
Age (years)	31.31 ± 9.38	27.92 ± 4.52	34.08 ± 5.70	0.03
Height (cm)	174.36 ± 5.33	177.50 ± 5.61	170.26 ± 4.56	< 0.01
Weight (kg)	73.88 ± 11.28	82.14 ± 10.81	71.98 ± 10.78	0.07
Body mass index (kg/m^2^)	24.19 ± 2.84	26.02 ± 2.78	24.85 ± 3.62	0.34

Notes: Data are represented as the mean ± standard deviation. *Analysis of variance test

### Pharmacokinetics

#### Effect of food on venadaparib in Korean and Caucasian subjects

**Fig. 2:**
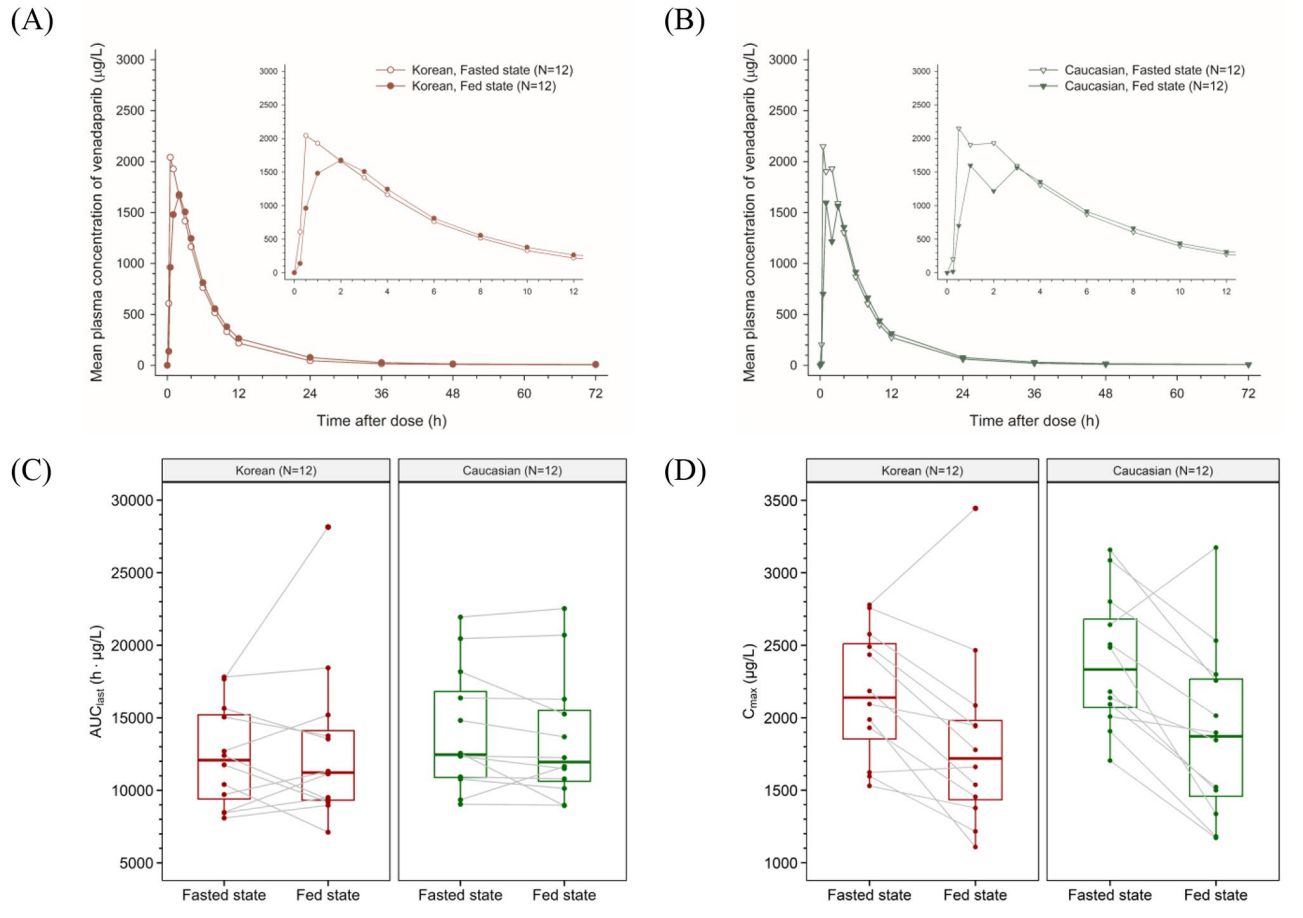
Effect of food on venadaparib in Korean and Caucasian subjects. Mean plasma concentration-time profiles of venadaparib after a single administration of venadaparib 80 mg in the fasted or fed states in (**A**) Korean and (**B**) Caucasian subjects. Boxplots of individual (**C**) AUC_last_ and (**D**) C_max_ of venadaparib after a single administration of venadaparib 80 mg in the fasted or fed states in each Korean and Caucasian subject. Notes: Boxplots represent the interquartile range (IQR) with whiskers extending from 1.5 IQR. Abbreviations: AUC_last_, the area under the plasma concentration-time curve from time zero to the last measurable time point; C_max_, maximum plasma concentration

The mean plasma concentration-time profiles of venadaparib following a single 80-mg dose showed delayed absorption with prolonged T_max_ and decreased C_max_ in the fed state compared with that in the fasted state (Fig. 2A, B). The median T_max_ values were prolonged from 0.5 h in the fasted state to 2 h in the fed state in Korean and Caucasian subjects (Table 2). The C_max_ in the fed state decreased by 18% in Koreans and 23% in Caucasians compared with that in the fasted state; however, the AUC_last_ values were similar between the two treatment groups (Fig. 2C, D; Table 2).

**Table 2:** Summary of pharmacokinetic parameters of venadaparib after a single administration of venadaparib 80 mg

Pharmacokinetic parameters	Fasted state (N = 12)	Fed state (N = 12)	Geometric mean ratio* (90% confidence interval)
Korean			
T_max_ (h)	0.50 (0.50–2.00)	2.00 (0.50–4.00)	.
C_max_ (μg/L)	2165.59 ± 446.08	1835.01 ± 637.11	0.82 (0.7457–0.9094)
AUC_last_ (h∙μg/L)	12347.13 ± 3514.30	12972.02 ± 5734.25	1.02 (0.9088–1.1339)
AUC_inf_ (h∙μg/L)	12453.18 ± 3565.66	13108.90 ± 5771.31	1.02 (0.9126–1.1360)
t_1/2_ (h)	9.06 ± 6.08	10.80 ± 5.10	.
CL/F (L/h)	6.92 ± 1.95	6.95 ± 2.29	.
V_d_/F (L)	83.84 ± 48.70	104.18 ± 57.31	.
Caucasian			
T_max_ (h)	0.50 (0.50–2.00)	2.00 (1.00–4.00)	.
C_max_ (μg/L)	2392.29 ± 464.48	1894.30 ± 602.49	0.77 (0.6871–0.8609)
AUC_last_ (h∙μg/L)	14091.32 ± 4285.15	13557.03 ± 4401.60	0.96 (0.9017–1.0186)
AUC_inf_ (h∙μg/L)	14202.62 ± 4352.38	13752.20 ± 4522.49	0.96 (0.9074–1.0260)
t_1/2_ (h)	9.32 ± 4.98	14.16 ± 7.16	.
CL/F (L/h)	6.10 ± 1.73	6.33 ± 1.78	.
V_d_/F (L)	72.52 ± 18.25	124.29 ± 71.28	.
Chinese			
T_max_ (h)	1.00 (0.50–2.00)	.	.
C_max_ (μg/L)	2390.12 ± 576.02	.	.
AUC_last_ (h∙μg/L)	14878.51 ± 4254.97	.	.
AUC_inf_ (h∙μg/L)	14981.87 ± 4276.66	.	.
t_1/2_ (h)	6.95 ± 4.18	.	.
CL/F (L/h)	5.84 ± 2.02	.	.
V_d_/F (L)	53.23 ± 20.51	.	.

Notes: Data are represented as the mean ± standard deviation, except for T_max_ represented as median (minimum–maximum). *Geometric mean ratio of fed to fasted states

Abbreviations: AUC_last_, the area under the plasma concentration-time curve (AUC) from time zero to the last measurable time point; AUC_inf_, AUC from time zero to infinity; C_max_, maximum plasma concentration; CL/F, apparent total clearance; t_1/2_, elimination half-life; T_max_, time to reach C_max_; V_d_/F, apparent volume of distribution

#### Effect of ethnicity on venadaparib among Korean, Caucasian, and Chinese subjects

**Fig. 3:**
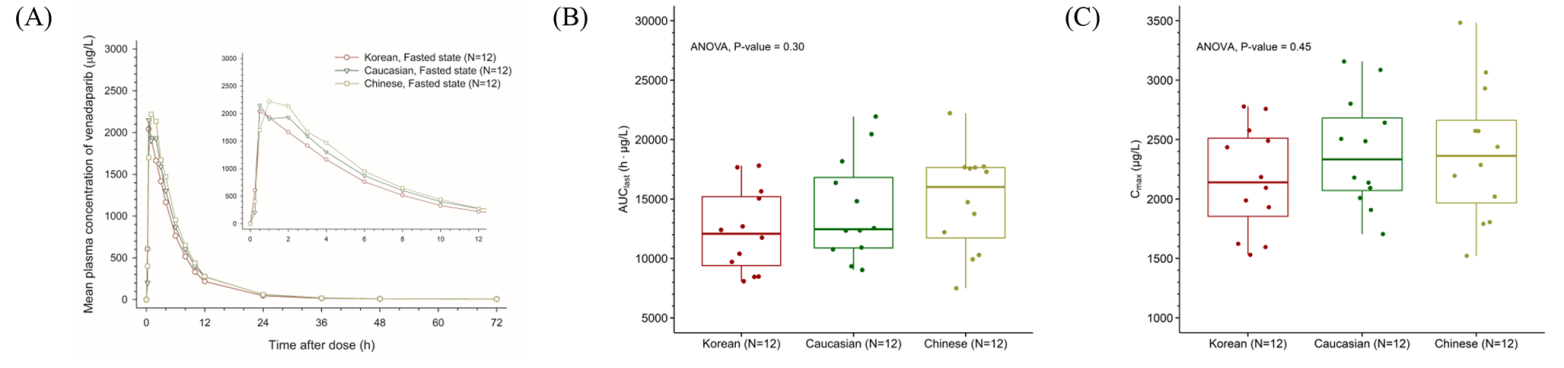
Effect of ethnicity on venadaparib among Korean, Caucasian, and Chinese subjects. (**A**) Mean plasma concentration-time profiles of venadaparib after a single administration of venadaparib 80 mg in the fasted state in Korean, Caucasian, and Chinese subjects. Boxplots of (**B**) AUC_last_ and (**C**) C_max_ of venadaparib after a single administration of venadaparib 80 mg in the fasted state in Korean, Caucasian, and Chinese subjects. Notes: Boxplots represent the interquartile range (IQR) with whiskers extending from 1.5 IQR. Abbreviations: AUC_last_, the area under the plasma concentration-time curve from time zero to the last measurable time point; C_max_, maximum plasma concentration

The mean plasma concentration-time profiles of venadaparib after a single administration of venadaparib 80 mg in the fasted state were similar among Korean, Caucasian, and Chinese subjects (Fig. 3A). The C_max_ and AUC_last_ values of venadaparib were comparable among the three ethnic groups, considering inter-subject variability (Table 2). No statistically significant differences were observed in the C_max_ and AUC_last_ values (*P*-values are 0.45 and 0.30, respectively) (Fig. 3B, C).

### Safety

In Koreans and Caucasians, nine treatment-emergent AEs (TEAEs) occurred in seven subjects in the fasted state, and 25 TEAEs occurred in 13 subjects in the fed state (Table 3). The most commonly observed TEAEs were nausea and decreased appetite. The number of subjects and events tended to increase in the fed state in Koreans, not Caucasians. In the fasted state, five, four, and three TEAEs occurred in four Koreans, three Caucasians, and two Chinese participants. The incidence of TEAEs was comparable among the three ethnic groups.

**Table 3:** Summary of treatment-emergent adverse events by system organ class and preferred term after a single administration of venadaparib 80 mg

System organ class Preferred term	Korean		Caucasian		Chinese
	Fasted state (N = 12)	Fed state (N = 13)	Fasted state (N = 12)	Fed state (N = 12)	Fasted state (N = 12)
Total	4 (33.3) [5]	6 (46.2) [16]	3 (25.0) [4]	7 (58.3) [9]	2 (16.7) [3]
Gastrointestinal disorders	3 (25.0) [4]	5 (38.5) [7]	1 (8.3) [1]	1 (8.3) [1]	1 (8.3) [1]
Nausea	1 (8.3) [1]	5 (38.5) [5]	1 (8.3) [1]	.	.
Dyspepsia	1 (8.3) [1]	1 (7.7) [2]	.	.	1 (8.3) [1]
Abdominal pain	1 (8.3) [1]	.	.	.	.
Epigastric discomfort	.	.	.	1 (8.3) [1]	.
Gingival pain	1 (8.3) [1]	.	.	.	.
Metabolism and nutrition disorders	.	3 (23.1) [3]	1 (8.3) [1]	2 (16.7) [2]	2 (16.7) [2]
Decreased appetite	.	3 (23.1) [3]	.	1 (8.3) [1]	1 (8.3) [1]
Hyperuricaemia	.	.	1 (8.3) [1]	1 (8.3) [1]	.
Hypertriglyceridaemia	.	.	.	.	1 (8.3) [1]
Eye disorders	.	1 (7.7) [3]	1 (8.3) [1]	.	.
Chalazion	.	.	1 (8.3) [1]	.	.
Eye pain	.	1 (7.7) [1]	.	.	.
Lacrimation increased	.	1 (7.7) [1]	.	.	.
Vision blurred	.	1 (7.7) [1]	.	.	.
Nervous system disorders	.	.	.	2 (16.7) [2]	.
Headache	.	.	.	1 (8.3) [1]	.
Lethargy	.	.	.	1 (8.3) [1]	.
Investigations	.	.	1 (8.3) [1]	1 (8.3) [1]	.
Blood bilirubin increased	.	.	1 (8.3) [1]	1 (8.3) [1]	.
Blood and lymphatic system disorders	.	.	.	1 (8.3) [1]	.
Eosinophilia	.	.	.	1 (8.3) [1]	.
General disorders and administration site conditions	.	.	.	1 (8.3) [1]	.
Thirst	.	.	.	1 (8.3) [1]	.
Infections and infestations	1 (8.3) [1]	.	.	.	.
Oral herpes	1 (8.3) [1]	.	.	.	.
Musculoskeletal and connective tissue disorders	.	1 (7.7) [1]	.	.	.
Rhabdomyolysis	.	1 (7.7) [1]	.	.	.
Respiratory, thoracic, and mediastinal disorders	.	1 (7.7) [1]	.	.	.
Rhinorrhoea	.	1 (7.7) [1]	.	.	.
Skin and subcutaneous tissue disorders	.	.	.	1 (8.3) [1]	.
Dermatitis contact	.	.	.	1 (8.3) [1]	.
Vascular disorders	.	1 (7.7) [1]	.	.	.
Hyperaemia	.	1 (7.7) [1]	.	.	.

Notes: Data are represented as the number of subjects (percentage of subjects) [number of events]

No serious AEs were observed, and all TEAEs were mild in severity except for contact dermatitis in one Caucasian subject in the fed state. The contact dermatitis was moderate; however, no causal relationship was observed with the study drug. Hypertriglyceridaemia was not an adverse drug reaction. All AEs were resolved.

No clinically significant changes were observed in the vital signs or 12-lead ECGs. The plasma concentrations of venadaparib did not prolong the QTcF and QTcB values, including changes from the baseline (Online Resource 1).

## Discussion

Understanding food-drug interactions and ethnic contributions to drug exposure and response is an important consideration in the drug development process [23, 24]. The intake of food or drinks can affect the PK profile of orally administered drugs by changing the physiology of the human gastrointestinal tract [23]. Ethnically diverse factors such as genetic, environmental, and physiological differences may also influence drug exposure and response [24]. This study was conducted on Korean, Caucasian, and Chinese subjects to evaluate the effects of food and ethnicity on venadaparib, a novel and selective PARP inhibitor. In Koreans and Caucasians, venadaparib absorption was delayed, with peak concentrations being reduced by approximately 20% after a high-fat meal; however, systemic exposure was similar to that in the fasted state. Additionally, the C_max_ and AUC_last_ values were comparable in the fasted states of the three ethnic groups, indicating no significant ethnic differences.

The mean plasma concentration-time profiles of venadaparib following a single venadaparib 80 mg dose showed delayed absorption with prolonged T_max_ and decreased C_max_ values in the fed state compared with those in the fasted state. However, the overall exposure to venadaparib, as measured by the AUC_last_ and AUC_inf_, demonstrated no significant difference between the two treatment groups, indicating comparable bioavailability from CL/F = dose/AUC_inf_. The tendency and extent of changes in the PK parameters observed with venadaparib were consistent with other PARP inhibitors, such as olaparib tablets and niraparib capsules [17, 25–28]. For olaparib tablets, the C_max_ value in the fed state decreased by 21% compared with that in the fasted state; however, AUC_last_ increased by 8% [25]. For niraparib capsules, the C_max_ value decreased by 27%, and the AUC_last_ value remained unchanged [25]. The relationship between exposure (time-varying average concentration) and efficacy was determined for talazoparib; however, inconclusive results were obtained for olaparib, niraparib, and rucaparib [25, 29]. The current labels of FDA-approved PARP inhibitors indicate that the tablets or capsules can be administered with or without food [30–33]. These results suggest that venadaparib can be administered with or without food, depending on the patient’s preference or individual tolerance.

Ethnic differences in the activities of the enzymes involved in drug metabolism lead to differences in drug exposure and responses. Each PARP inhibitor has different metabolic pathways; however, information on changes in PKs and PDs based on ethnicity is limited due to the lack of diversity in clinical trial populations [25, 34–36]. Venadaparib is primarily metabolized by cytochrome P450 3A4 based on the in vitro phenotyping study (data on file), which differs in the frequencies of clinically relevant variant alleles among ethnic groups and may contribute to ethnic differences in drug exposure and response [34]. However, no statistically significant differences were observed in the C_max_ (*P*-value = 0.45) and AUC_last_ (*P*-value = 0.30) values of venadaparib among Korean, Caucasian, and Chinese subjects in our study. We expect that venadaparib would show similar responses among ethnic groups; however, this needs confirmation by further clinical trials.

The FDA guidance for assessing the effects of food on drugs suggests that the highest clinically recommended dose should be selected as the dose for the food effect study unless safety concerns necessitate a lower dose [20]. This study was designed with 80 mg of venadaparib, which is half the potential recommended phase 2 dose, considering that anticancer agents are administered to healthy volunteers. The safety results showed that a single venadaparib 80 mg dose was safe and well-tolerated in healthy volunteers in both fasted and fed states. Meanwhile, in the safety profile of Koreans, the number of subjects and events of nausea and decreased appetite tended to increase in the fed state than in the fasted state; however, this was not observed in Caucasians. Although nausea and decreased appetite are commonly known AEs of PARP inhibitors [17], these trends in the incidence of TEAEs were not significantly associated with venadaparib exposure altered by food (Online Resource 2). We speculate that Korean subjects, who typically consume less fatty diets than Western diets, exhibited this trend as they consumed an unusually fatty and greasy breakfast for this study [37–40]. In addition to the dietary habits, ethnic differences in safety may be influenced by multiple factors, including pathophysiological, environmental, cultural, and socio-politico-economic differences [24, 41, 42]. Therefore, further pooled patient population analyses may be needed to compare the safety and tolerability of venadaparib in different ethnic groups.

## Conclusion

The overall systemic exposure to venadaparib was not significantly affected by the high-fat meal, despite delayed absorption with a decreased C_max_ value in the fed state compared with that in the fasted state. The PK profiles were comparable among the Korean, Caucasian, and Chinese subjects. A single administration of venadaparib 80 mg was safe and well-tolerated in both the fasted and fed states.

Therefore, venadaparib can be administered with or without food, depending on the patient’s preference or individual tolerance. Additionally, venadaparib is expected to show similar responses among ethnic groups, suggesting little need for dose adjustment according to ethnic groups.

### Electronic supplementary material

Below is the link to the electronic supplementary material.

Supplementary Material 1

Supplementary Material 2

## Data Availability

Data supporting the findings of this study are available from the corresponding author, Kyung-Sang Yu, upon reasonable request.
