# Supplementary material for: Effects of food and ethnicity on the pharmacokinetics of venadaparib, a next-generation PARP inhibitor, in healthy Korean, Caucasian, and Chinese male subjects
Source: Invest New Drugs. 2023 Dec 15;42(1):80–8. doi: 10.1007/s10637-023-01405-z (PMC10891214; doi:10.1007/s10637-023-01405-z)
Supplement: Supplementary file 1 — Supplementary Material 1 [file 10637_2023_1405_MOESM1_ESM.docx]

| (A) | 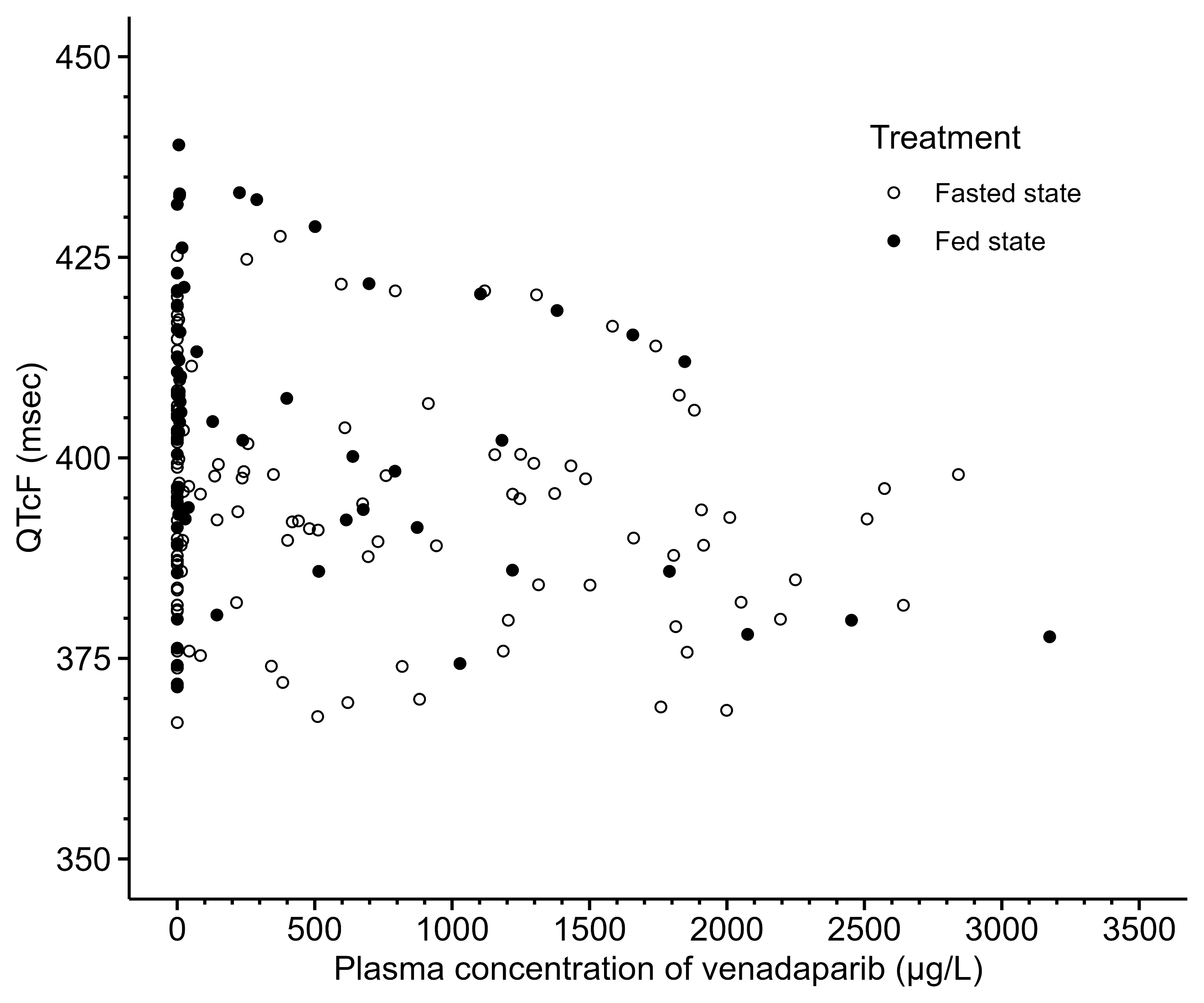 | (B) | 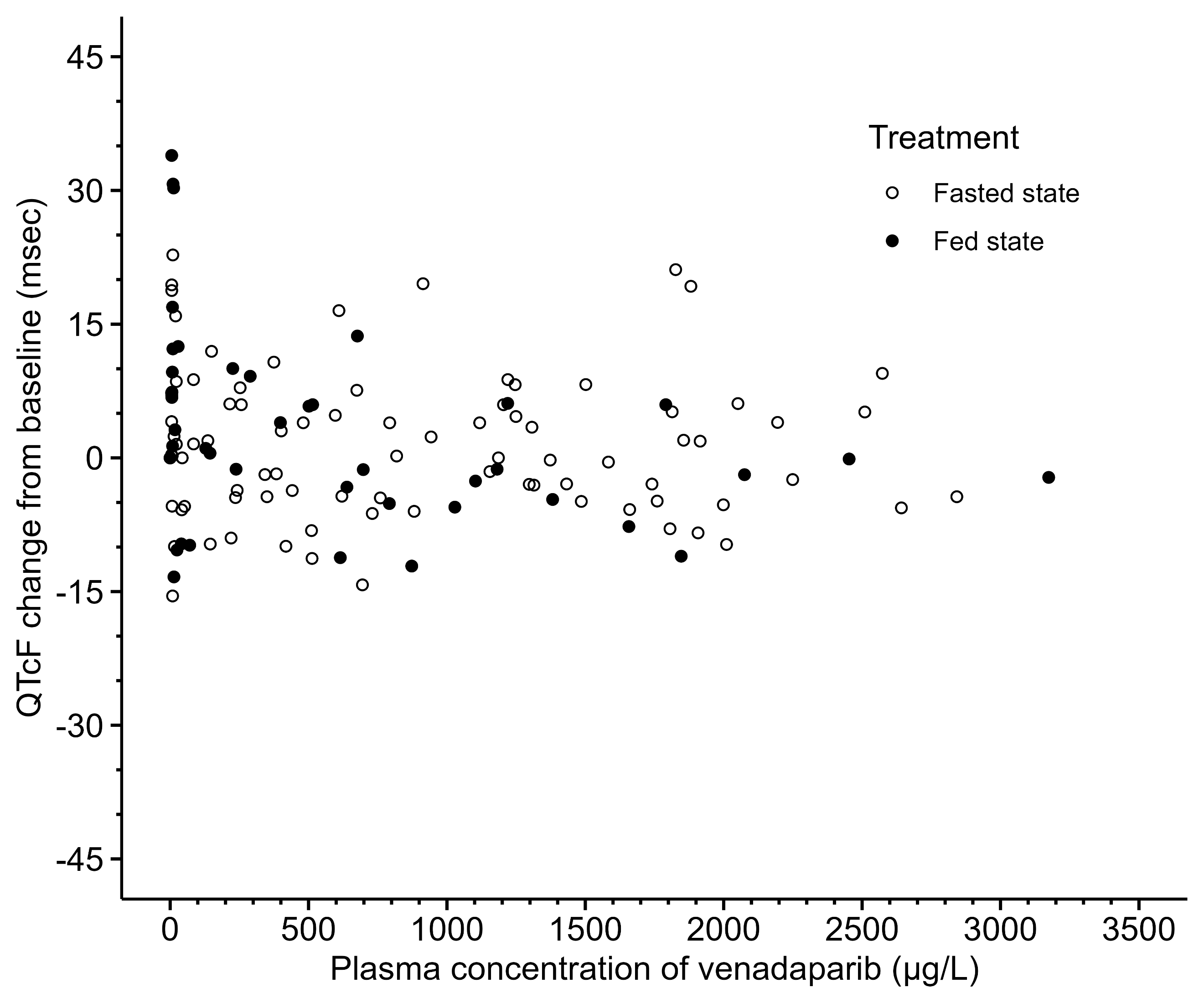 |
| --- | --- | --- | --- |
| (C) | 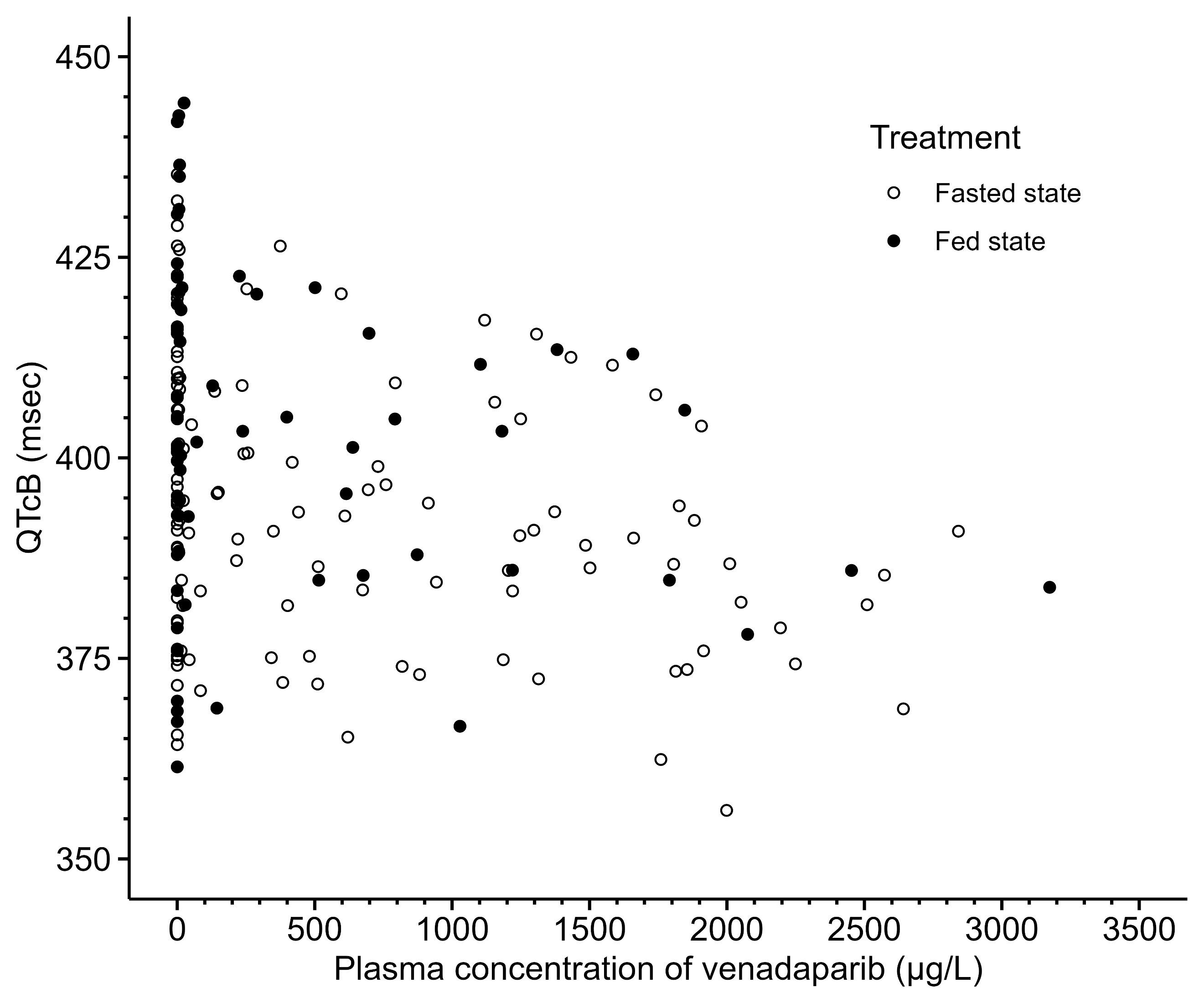 | (D) | 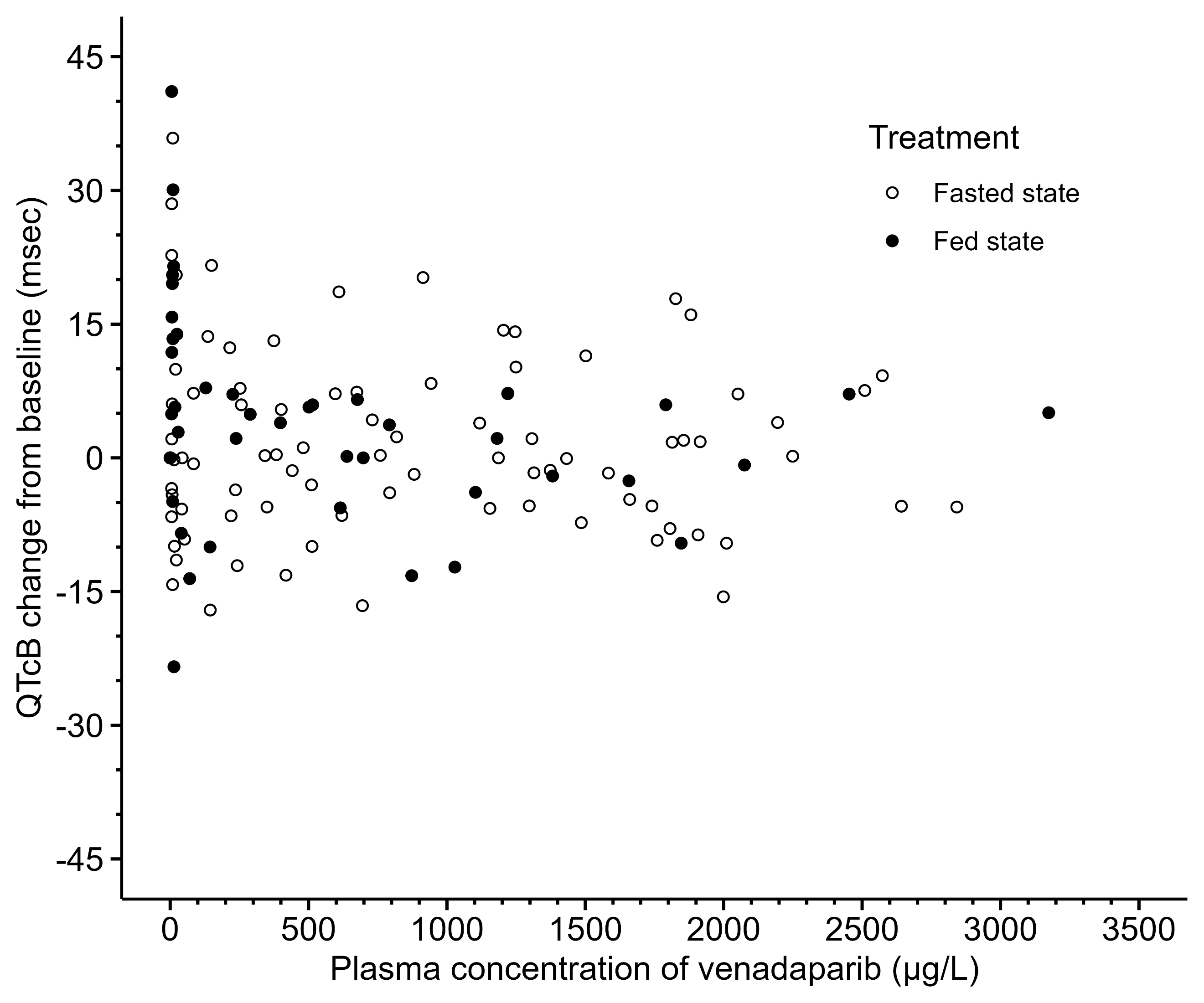 |

Fig. S1 (A) QTcF, (B) change from baseline in QTcF, (C) QTcB, and (D) change from baseline in QTcB versus plasma concentration of venadaparib after a single administration of venadaparib 80 mg in Korean, Caucasian, and Chinese subjects. Abbreviations: QTcF, Fridericia’s corrected QT interval; QTcB, Bazett’s corrected QT interval
