# Supplementary material for: Effects of food and ethnicity on the pharmacokinetics of venadaparib, a next-generation PARP inhibitor, in healthy Korean, Caucasian, and Chinese male subjects
Source: Invest New Drugs. 2023 Dec 15;42(1):80–8. doi: 10.1007/s10637-023-01405-z (PMC10891214; doi:10.1007/s10637-023-01405-z)
Supplement: Supplementary file 2 — Supplementary Material 2 [file 10637_2023_1405_MOESM2_ESM.docx]

| (A) | 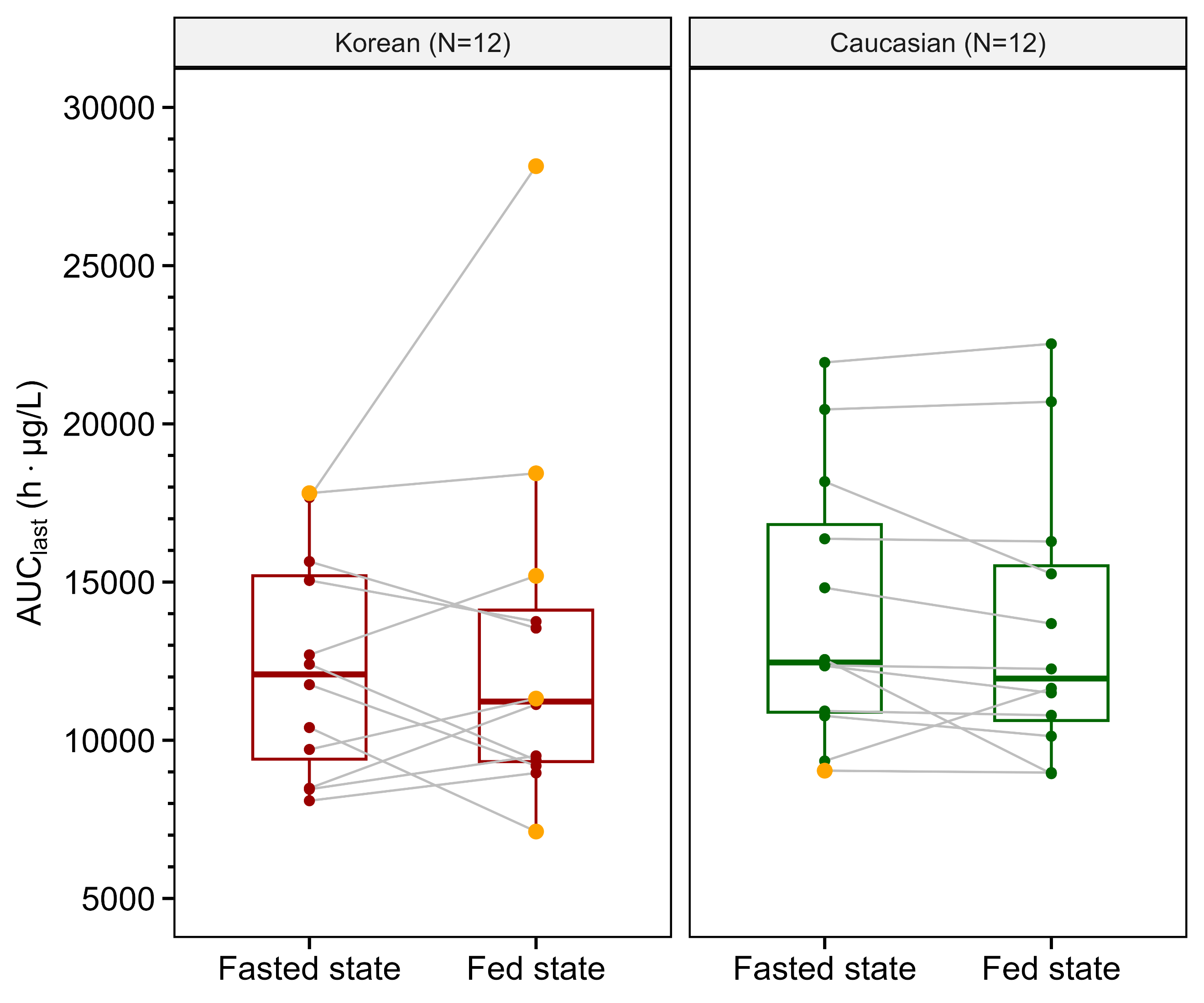 | (B) | 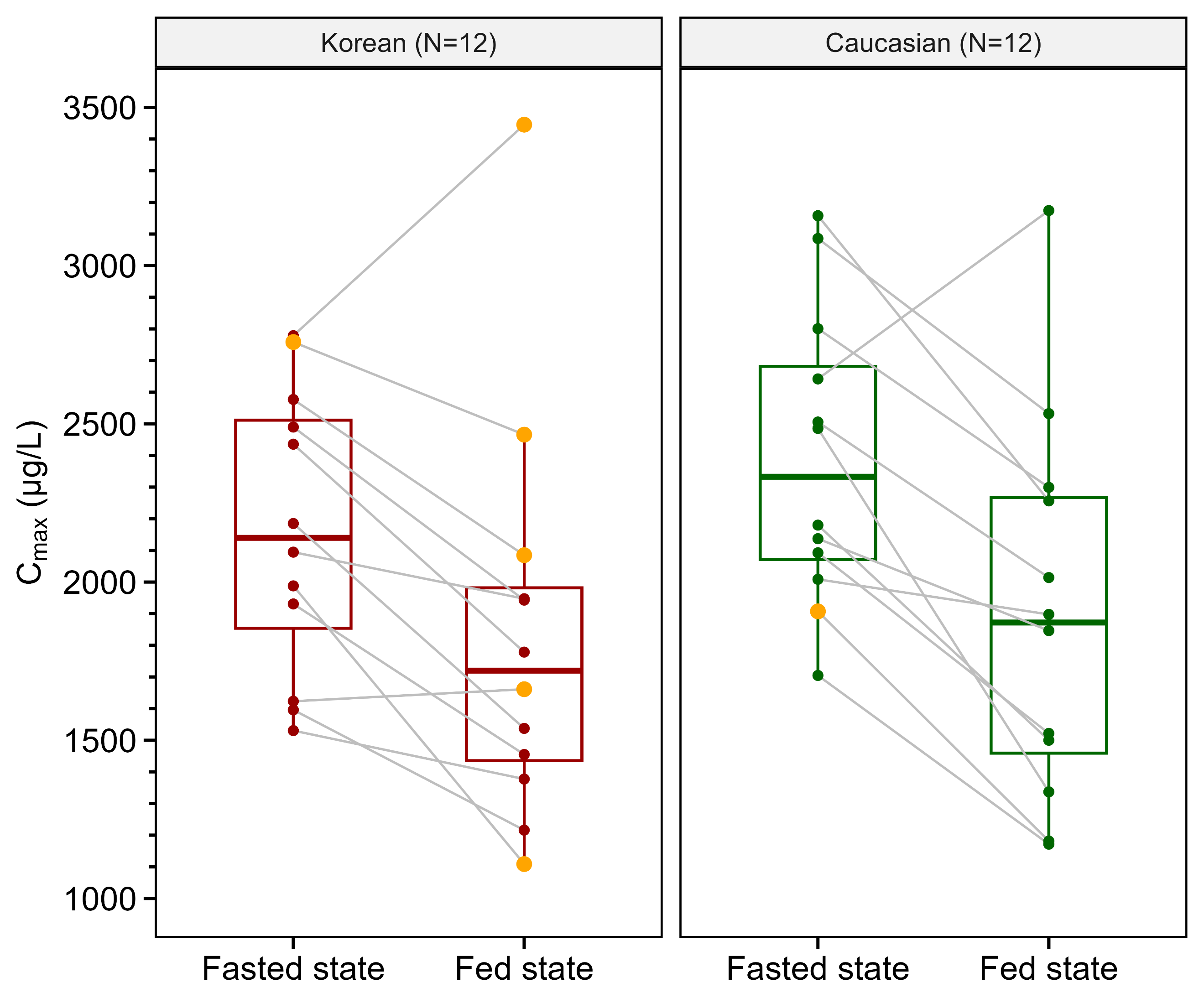 |
| --- | --- | --- | --- |
| (C) | 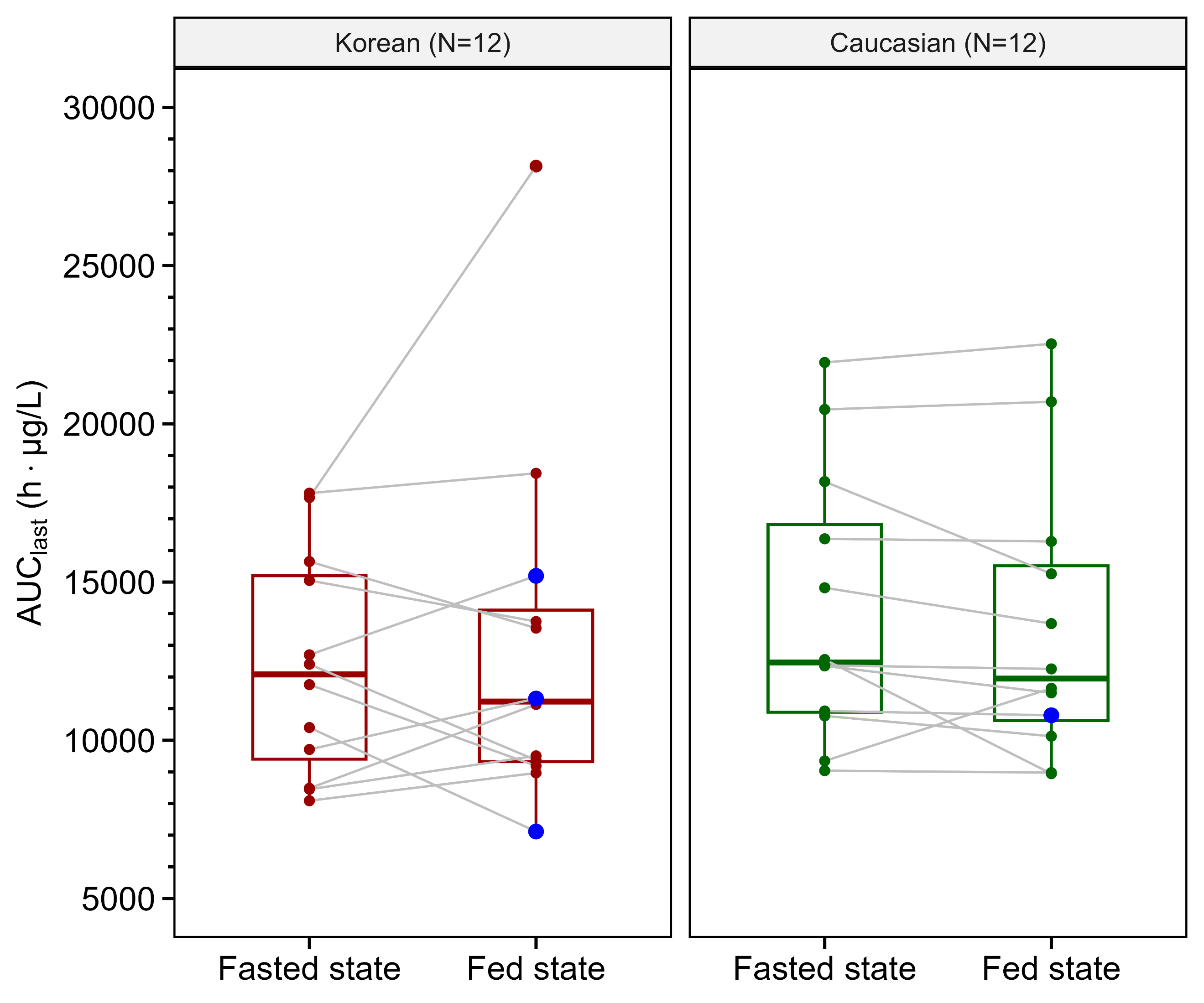 | (D) | 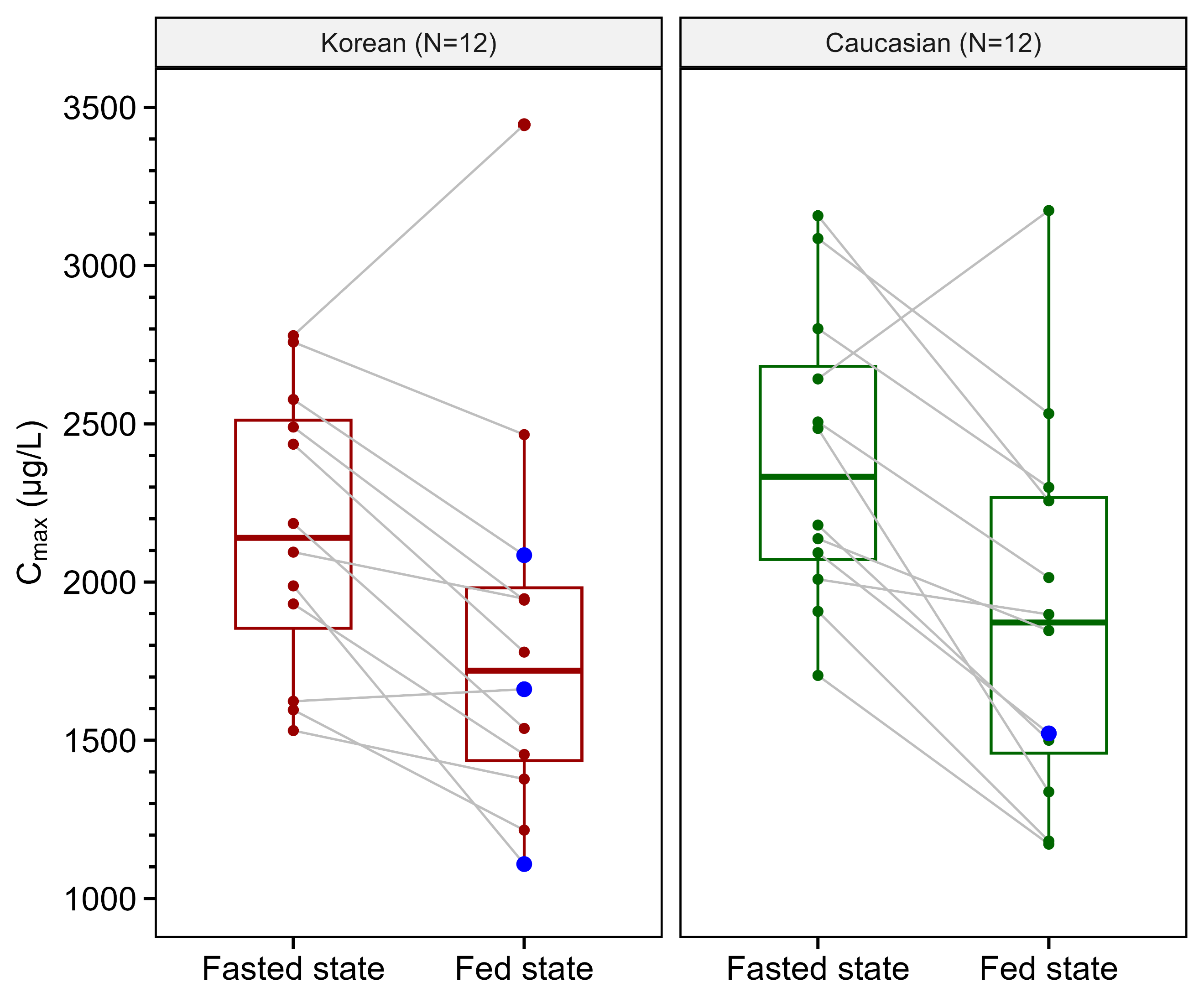 |

Fig. S2 Relationship between pharmacokinetic parameters (AUC_last_ and C_max_) and treatment-emergent adverse events (nausea and decreased appetite) by the effect of food on venadaparib in Korean and Caucasian subjects. Notes: Boxplots represent the interquartile range (IQR) with whiskers extending from 1.5 IQR. Orange points represent subjects who reported nausea, and blue points represent subjects who reported decreased appetite. Abbreviations: AUC_last_, the area under the plasma concentration-time curve from time zero to the last measurable time point; C_max_, maximum plasma concentration
